# Prevalence and factors associated with trajectories of antenatal depression: a prospective multi-center cohort study in Chengdu, China

**DOI:** 10.1186/s12884-023-05672-9

**Published:** 2023-05-17

**Authors:** Xiuhua Huang, Ying Wang, Yuqiong Wang, Xiujing Guo, Ling Zhang, Wenxia Wang, Jing Shen

**Affiliations:** 1 Department of Pediatric Surgery, Chengdu Women’s and Children’s Central Hospital, School of Medicine, University of Electronic Science and Technology of China, Chengdu, China; 2 Department of Child Healthcare, Chengdu Women’s and Children’s Central Hospital, School of Medicine, University of Electronic Science and Technology of China, Chengdu, China; 3 Department of Nursing, Chengdu Women’s and Children’s Central Hospital, School of Medicine, University of Electronic Science and Technology of China, Chengdu, China; 4 Department of Obstetrics, West China Second University Hospital, Sichuan University/ West China School of Nursing, Sichuan University, Chengdu, China; 5 Key Laboratory of Birth Defects and Related Diseases of Women and Children (Sichuan University), Ministry of Education, Chengdu, China; 6 Pediatric Intensive Care Unit, Chengdu Women’s and Children’s Central Hospital, School of Medicine, University of Electronic Science and Technology of China, Chengdu, China; 7 Department of Outpatient, Chengdu Women’s and Children’s Central Hospital, School of Medicine, University of Electronic Science and Technology of China, Chengdu, China; 8 Department of Obstetrics, Chengdu Women’s and Children’s Central Hospital, School of Medicine, University of Electronic Science and Technology of China, Chengdu, China

**Keywords:** Antenatal depression, EPDS, Trajectory model, Associated factors

## Abstract

**Background:**

Antenatal depression (AD) is a major depressive disorder during pregnancy, which may lead to devastating sequelae for the expectant mothers and infants. This study aimed to investigate the prevalence, to analyze trajectory model based on EPDS score**,** and to explore the influence factors of AD among pregnant women in Chengdu, China.

**Methods:**

Participants from four maternity hospitals in Chengdu, China were recruited when they had their first pregnancy medical check-up during March 2019 to May 2020. All participants were required to fill in Edinburgh Postnatal Depression Scale Chinese version (EPDS) once during three trimesters and provided information about their health status, social-demographic etc. The trajectory model, chi-square test and multivariate binary logistic regression were used to analyze all collected data.

**Results:**

A total of 4560 pregnant women were recruited, while 1051 women completed the study. The prevalence of depression symptoms during the first, second and third trimesters were 32.92% (346/1051), 19.79% (208/1051) and 20.46% (215/1051) respectively. According to the results of the latent growth mixture modeling, the trajectory model of three categories based on EPDS score were identified in this study: low-risk group (38.2%, 401/1051), medium-risk group (54.8%, 576/1051) and high-risk group (7%, 74/1051). Good marital relationship (*P* = 0.007, OR = 0.33, 95% *CI* 0.147 ~ 0.74), good relationship with parents-in-law (*P* = 0.011, OR = 0.561, 95% *CI* 0.36 ~ 0.874), planned pregnancy (*P* = 0.018, OR = 0.681, 95% *CI* 0.496 ~ 0.936) were the protective factors while lower education level (*P* = 0.036, OR = 1.355, 95% *CI* 1.02 ~ 1.799), fear about dystocia (*P* = 0.0, OR = 1.729, 95% *CI* 1.31 ~ 2.283), recent major negative life events (*P* = 0.033, OR = 2.147, 95% *CI* 1.065 ~ 4.329) were the risk factors of medium-risk group. Good marital relationship (*P* = 0.005, OR = 0.2, 95% *CI* 0.065 ~ 0.615), good relationship with parents-in-law (*P* = 0.003, OR = 0.319, 95% *CI* 0.15 ~ 0.679) were also protective factors of high-risk group, but the risk factors for high-risk group were medical history (*P* = 0.046, OR = 1.836, 95% *CI* 1.011 ~ 3.334), pregnancy complications (*P* = 0.022, OR = 2.015, 95% *CI* 1.109 ~ 3.662), worry about dystocia (*P* = 0.003, OR = 2.365, 95% *CI* 1.347 ~ 4.153), recent major negative life events (*P* = 0.011, OR = 3.661, 95% *CI* 1.341 ~ 9.993). No protective or risk factors were identified for low-risk group.

**Conclusion:**

Even the incidence and levels of depression in the first trimester of pregnancy were the highest, the probability of pregnancy women get depression during gestation period were higher than other population. Therefore, it’s important to monitor the psychological status of pregnant women during the whole pregnancy, especially in the first trimester. The study suggested a good partner relationship and good relations with parents-in-law both protected pregnant women from depression and promoted the well-being of mothers and children.

## Introduction

Antenatal depression (AD) is one of the most prevalent psychiatric disorders, which may lead to devastating impacts on both mothers and infants’ health [1]. The pregnant women who has AD are at high risk for preeclampsia, cesarean section, prolonged delivery process, dystocia, postpartum hemorrhage [2–4], postpartum depression and maternal suicide [4–6]. For infants whose mother once had AD, they are more likely to have a higher incidence of low birth weight, premature delivery, neonatal death [4, 7–12], delayed fetal growth and brain development [13–15]. It can also have a significant persistent impact on their cognitive, behavioral and emotional development [16].

The recent meta-analysis reported the prevalence of AD is 18.1% in high-income countries (HICs), 24.2 to 30.8% in Low- and Middle-Income Countries (LMICs)[17]. Studies covered Asian area found that prevalence of AD is different across countries: 14 to 16.3% in Japan [18], 29.4% in South-east Asian region [19], and 4 to 46.11% in China [20–22] which showed a big variation. Hence it is necessary to establish a more reliable morbidity data of AD in China. Beyond that, AD may occur in any trimester during pregnancy. Yin’s systematic reviews and meta-analyses showed the incidence rate of AD is 21.2% during the first trimester, 15.8% during the second trimester, 18.9% during the third trimester [19].

Relevant factors from studies were summarized into the following major categories: pregnant.women’s demographic characteristics (such as ethnicity, marital status, age, education, employment); psycho-social factors (social and family support, experience of violence, and stressful life events); health-related factors (such as history of depression, history of abortion, chronic medical conditions, and pregnancy complications); as well as lifestyle and cognitive aspect (smoking, drinking, planned pregnancy) [20, 21]. Compared with western women, Chinese women may be more family-oriented and easier affected by family relationship and family support [22]. Lau et al. [23] revealed marital status, family interpersonal relationships, and social support might contribute to AD patients living in the city of Chengdu, China.

Most of studies only investigated the prevalence of AD when pregnant women during her third trimester, while quite few studies cared about the situation of AD in first and second trimester. There were little studies tracked the incidence of AD across first to third trimester, especially the trajectory of depression during pregnancy which may help to understand the development of AD.

Thus, the current study aimed to establish the prevalence and trajectory model of AD based on EPDS score across the whole pregnancy. Meanwhile to explore the possible influence factors of AD among pregnant women. Ultimately, this study also aimed to screen the pregnant women who have depression and try to provide them with intervention, as well as set up measurements for improving the mental health of pregnant women in Chengdu across all trimesters.

## Methods

### Study design and participants

This study was a prospective multi center cohort survey. Multi-stage stratified convenient sampling was adopted in the research. Stage 1: From the official website of Chengdu Municipal Health Commission, four maternity hospitals at different levels (including one ministerial, one provincial, one municipal and twenty district hospitals) were selected by using the method of stratified convenient sampling. Stage 2: We recruited pregnant women by posters who attended their first pregnancy medical check-up (all during their first trimester) and had follow-up at four selected antenatal maternity hospitals between March 2019 and May 2020, by using the method of convenient sampling. Inclusion criteria: 1) women diagnosed with pregnancy (12^+6^ weeks and before); 2) being able to listen, read and write. Exclusion criteria: 1) pregnant with a history of mental diseases or mental disorders; 2) failing to complete the study; 3) obvious errors or missing data more than 20% in the survey.

### Measurement

All participants as they diagnosed with pregnancy in the first stage would be invited to attend the study. And each woman completed the written questionnaires three times (in their first trimester, second trimester and third trimester of the pregnancy, respectively) with the researcher in a private consultation room. An interval for each collection was at least four weeks. The questionnaires consisted of two parts: I. General information (self-designed questions): (a) social-demographic data included age, education, marriage and occupation, etc.; (b) Social support factors in recent three months included marital relationship, relations with parents-in-law, household income, recent major life events and taking care of children, etc.; (c) Obstetric information included disease history, times of pregnancy, times of delivery, appetite, sleep and pregnancy complications, etc.; (d) Cognitive factors included pregnancy plan, fear of dystocia, etc.; II. Instrument of assessment of AD: Edinburgh Postpartum Depression Scale (EPDS) was developed by Cox et al. [24] in 1987, and translated and back-translated from English to Chinese and standardized by Wang et al. [25] in 2009. It is widely used as a screening instrument for maternal depression in Mainland China. EPDS has 10 items and each item is scored on a 4-point scale (0 to 3). A cutoff score of 9.5 or higher has been suggested as a positive screen for clinic depression (higher score indicating more depressive symptoms), with the reliability of 0.76 and the validity of 0.93, respectively.

### Ethical considerations

The study was approved by the human research Ethics Committee of four maternity hospitals (No. 2019002). During the survey, the main concepts and purposes of the study were elucidated to the participants, and the consent was obtained. The subjects were informed that the questionnaire was anonymous and they could abandon the survey at any time. The raw data were saved with password and data is only used for research purpose.

### Statistical analysis

EpiData software version 3.1 was used to establish the electronic database, and logical correction was conducted after data entered by two persons. Categorical variables were expressed as frequencies and percentages. Non-normal continuous variables were expressed as median and interquartile range (IQR). Mean and standard deviation (SD) were used to describe the continuous variables with normal distribution. The chi-square test, the Fisher’s exact test, the Mann–Whitney U-tests, and the Student’s t-test were used to compare the differences between groups. Multivariate binary logistic regression was used to analyze the multiple influencing factors. In this study, latent growth mixture modeling was used to analyze the trajectories based on the EPDS scores of pregnant women and full information maximum likelihood estimation was used to fit the unbalanced data in the analysis sample. The best model was selected according to Bayesian information criterion (BIC). The expected number of people in each group should account for no less than 5% of the total number of people. All the analyses were performed using the SAS software version 9.4 (SAS Institute Inc., Cary, NC, United States). A *p*-value of less than 0.05 was considered statistically significant.

## Results

**Fig. 1:**
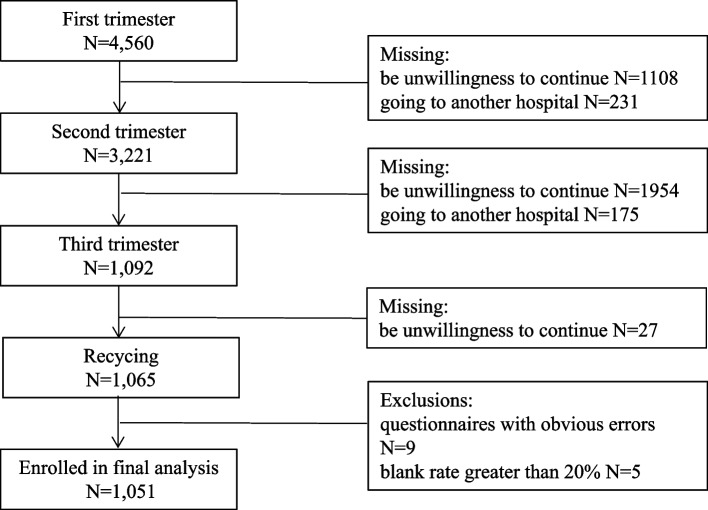
Flow diagram of the study

The flowchart represented the whole procedure of data collecting was presented in Fig. 1. The age of the subjects in this group was 20 ~ 41 years old, with an average of 28.94 ± 3.81 years old. The first trimester was 4 ~ 12^+ 6^ weeks, and the median was 12 weeks; The second trimester was 17 ~ 27^+ 6^ weeks, with a median of 24 weeks. The third trimester was 30 ^+ 2^ ~ 40 weeks, with a median of 35 weeks.

**Fig. 2:**
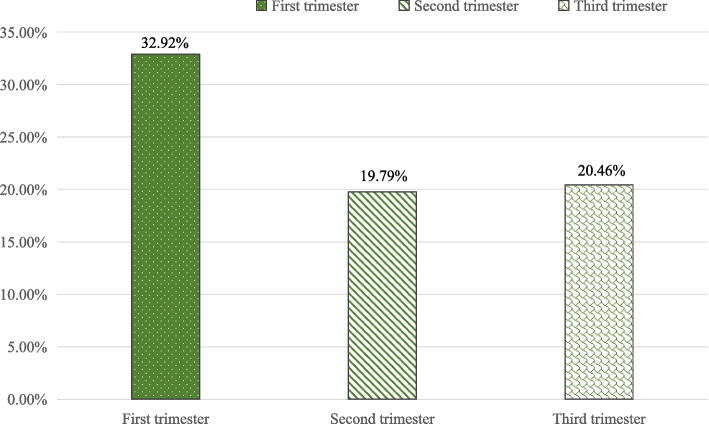
The Prevalence of AD at different trimesters of pregnancy

**Fig. 3:**
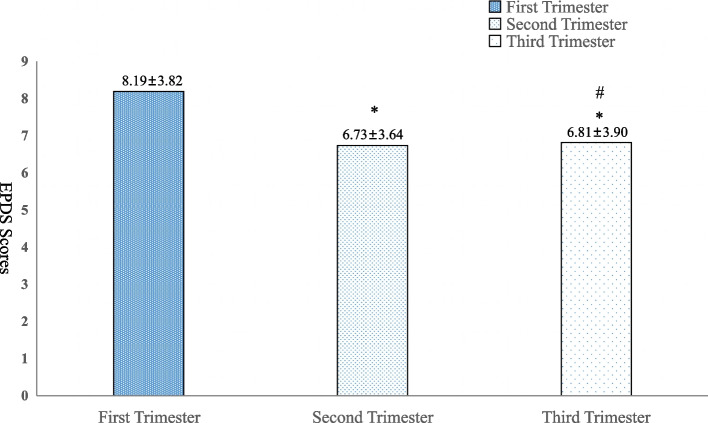
The scores of EPDS in different trimesters(X ±s). *P＜0.05 EPDS Scores of First Trimester vs. EPDS Scores of Second/ Third Trimester; #*P*＞0.05 EPDS Scores of Second Trimester vs. EPDS Scores of Third Trimester

The results showed that a total of 480 cases (45.67%) were screened for depression in at least a certain period of the entire stages of pregnancy in parallel screening. Among these data, there were 346, 208 and 215 pregnant women (32.92%, 19.79% and 20.46%) identified as depression in the first, second and third trimester respectively (Fig. 2). The incidence and levels of depression in the first trimester of pregnancy were the highest among the three trimesters of pregnancy. The detail of EPDS score in different trimesters were presented in Fig. 3.

**Fig. 4:**
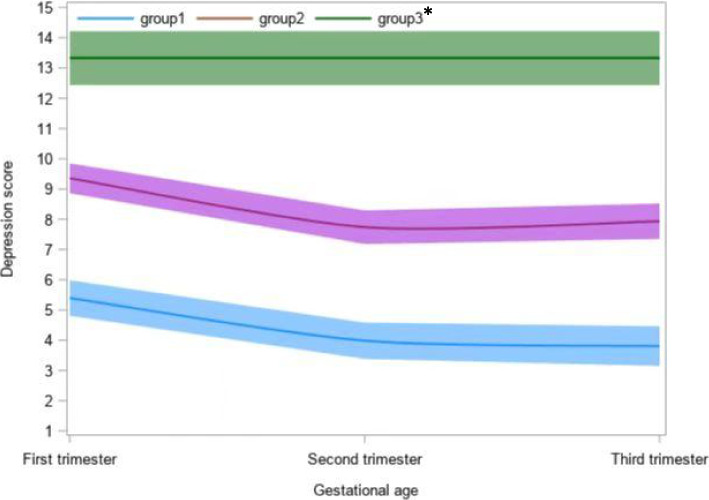
Trajectory model of depression scores during three trimesters of pregnancy. *group 1 was named low risk group; group 2 was named medium risk group; group 2 was named high risk group

According to the results of the latent growth mixture modeling, the trajectory model of three categories was the best (Table 1). The depression score tracks of pregnant women in this study population were identified as three categories: low risk group (38.2%, 401/1051), medium risk group (54.8%, 576/1051) and high-risk group (7%, 74/1051) (Fig. 4). Pregnant women of high-risk may have prolonged depression situation more than two trimesters.

**Table 1:** Bayesian information criterion values of trajectory models limited to different number of groups

Groups in Trajectory Modeling, N	Bayesian information criterion	Log(2ΔBayesian information criterion)	Expected proportion of the number of people in each group to the total number of people (%)			
			Group 1	Group 2	Group 3	Group 4
1	-8634.08		100.00			
2	-8351.6	564.96	63.26	36.74		
3	-8258.93	173.94	38.24	53.43	8.33	
4	-8229.37	70.52	19.89	54.88	23.72	1.51^a^

^a^The expected number of people accounts for less than 5% of the total number of people

Compared with "low risk" group, the "medium risk" group and the "high risk" group had significant differences in education level, pregnancy thoughts, medical history, pregnancy complications, relationship with parents-in-law, marital relationship and major negative life events during pregnancy (*P* < 0.05). And the medium risk group had significant differences in planned pregnancy (*P* = 0.021). The details were shown in Table 2.

**Table 2:** Univariate analysis of low risk group compared with medium risk group, with high risk group[N(%)]

Variables	Medium risk group(*N* = 576)	Low risk group(*N* = 401)	χ^2^/P	High risk group(*N* = 74)	Low risk group(*N* = 401)	χ^2^/P
Level of education			8.47/.014			7.177/.028
Secondary school and below	108(58.1)	78(41.9)		22(22)	78(78)	
University and above	422(57.7)	310(42.3)		47(86.8)	310(13.2)	
Others(specialist, etc.)	42(77.8)	12(22.2)		5(29.4)	12(70.6)	
Marital status			.402/.526			1/1
Married	565(58.7)	398(41.3)		73(15.5)	398(84.5)	
Unmarried/Divorced/Widowed	8(72.7)	3(27.3)		1(25)	3(75)	
Personal work			2.69/.26			2.53/.283
Full-time	437(57.9)	318(42.1)		54	318	
Part-time	19(55.9)	15(44.1)		2	15	
No work	119(59)	66(41)		18	66	
Family income(yuan/per person/month)			2.05/.561			2.29/.514
< 2000	75(60.5)	49(39.5)		11(18.3)	49(81.7)	
2001–3000	89(59.7)	60(40.3)		15(20)	60(80)	
3001–4000	118(62.4)	71(37.6)		12(14.5)	71(85.5)	
> 4000	284(56.8)	216(43.2)		34(13.6)	216(86.4)	
History of childbirth			1.13/.287			.007/.935
No	528(58.5)	374(41.5)		69(15.6)	374(84.4)	
Yes	48(64.9)	26(35.1)		5(16.1)	26(83.9)	
Planned pregnancy			7.77/.021			4.15/.126
Planned pregnancy	411(56.6)	315(43.4)		50(13.7)	315(86.3)	
Unplanned pregnancy	149(64.8)	81(35.2)		23(22.1)	81(77.9)	
others	14(77.8)	4(22.2)		1(20)	4(80)	
Pregnancy thoughts			7.49/.024			7.64/.022
Mission	44(66.7)	22(33.3)		11(33.3)	22(66.7)	
Burden	34(75.6)	11(24.4)		2(15.4)	11(84.6)	
Happy thing	494(57.5)	365(42.5)		57(13.5)	365(86.5)	
The relationship with parents-in-law			16.33/0			23.19/0
Good	473(56.4)	366(43.6)		53(37.5)	366(62.5)	
General /bad	103(74.6)	35(25.4)		21(12.6)	35(87.4)	
The marital relationship			15.59/0			31.33/0
Good	531(57.5)	393(42.5)		62(13.6)	393(86.4)	
General /bad	45(84.9)	8(15.1)		12(60)	8(40)	
The father desired sex of the baby (male)			.002/.961			3.63/.057
Yes	58(59.2)	40(40.8)		13(24.5)	40(75.5)	
No	518(58.9)	361(41.1)		61(14.5)	361(85.5)	
Recent major negative life events			9.02/.003			22.63/0
Yes	41(78.8)	11(21.2)		10(47.6)	11(52.4)	
No	534(57.8)	390(42.2)		63(13.9)	301(86.1)	
Medical history			7.34/.007			6.32/.012
Yes	356(62.6)	213(37.4)		51(19.3)	213(80.7)	
No	220(53.9)	188(46.1)		23(10.9)	188(89.1)	
Pregnancy complications			4.92/.027			5.36/.021
Yes	323(62.2)	196(37.8)		47(19.3)	196(80.7)	
No	253(55.2)	205(44.8)		27(11.6)	205(88.4)	
Fear about dystocia			19.31/0			19.83/0
Yes	260(67.5)	125(32.5)		43(25.6)	125(74.4)	
No	316(53.4)	276(46.6)		31(10.1)	276(89.9)	

This study performed a binary logistic regression model for the single factors that were statistically significant. The results indicated that compared to low risk group, Good marital relationship (*P* = 0.007, OR = 0.33, 95% CI 0.147 ~ 0.74), good relationship with parents-in-law (*P* = 0.011, OR = 0.561, 95% *CI* 0.36 ~ 0.874), planned pregnancy (*P* = 0.018, OR = 0.681, 95% *CI* 0.496 ~ 0.936) were the protective factors of "medium risk" group, low education level (*P* = 0.036, OR = 1.355, 95% *CI* 1.02 ~ 1.799), fear about dystocia (*P* = 0.0, OR = 1.729, 95% *CI* 1.31 ~ 2.283), recent major negative life events (*P* = 0.033, OR = 2.147, 95% *CI* 1.065 ~ 4.329) were the risk factors of the "medium risk" group. Good marital relationship (*P* = 0.005, OR = 0.2, 95% *CI* 0.065 ~ 0.615), good relationship with parents-in-law (*P* = 0.003, OR = 0.319, 95% *CI* 0.15 ~ 0.679) were the protective factors of the "high-risk" group, medical history (*P* = 0.046, OR = 1.836, 95% *CI* 1.011 ~ 3.334), pregnancy complications (*P* = 0.022, OR = 2.015, 95% *CI* 1.109 ~ 3.662), worry about dystocia (*P* = 0.003, OR = 2.365, 95% *CI* 1.347 ~ 4.153), recent major negative life events (*P* = 0.011, OR = 3.661, 95% *CI* 1.341 ~ 9.993) were the risk factors of "high risk" group. The details were shown in Table 3.

**Table 3:** Multivariate analysis of low risk group compared with medium risk group, with high risk group

Variables	Medium risk group VS Low risk group Non depression group (576:401)		High risk group VS Low risk group(74:401)	
	P	OR(95%Cl)	P	OR(95%Cl)
Medical history	.053	.766(.584, 1.004)	.046	1.836(1.011, 3.334)
Pregnancy complications	.168	.806(.594, 1.095)	.022	2.015(1.109, 3.662)
The relationship with parents-in-law	.011	.561(.36, .874)	.003	.319(.15, .679)
The marital relationship	.007	.33(.147, .74)	.005	.2(.065, .615)
Recent major negative life events	.033	2.147(1.065, 4.329)	.011	3.661(1.341, 9.993)
Fear about dystocia	0	1.729(1.31, 2.283)	.003	2.365(1.347, 4.153)
Level of education	.036	1.355(1.02, 1.799)	.956	1.017(.554, 1.867)
Pregnancy thoughts	.101	.686(.437, 1.076)	.336	.653(.274, 1.556)
Planned pregnancy	.018	.681(.496, .936)	/	/

## Discussion

### The occurrence of antenatal depression at three trimesters during pregnancy

Depression may occur in any stage of pregnancy, however r the incidence and levels of depression were the highest in the first trimester of pregnancy (32.92%, 346/1051). In addition, the prevalence of depression symptoms during the second and third trimesters were 19.79% (208/1 051) and 20.46% (215/1 051), respectively. The finding of this study was in line with the study conducted by Lee [26], which found that women scoring depression in the first trimester was significantly higher than any other time. There might be several reasons. Firstly, pregnant women might suffer from discomfort or severe pregnancy sickness such as first-trimester nausea, extreme form of morning sickness such as Hyperemesis Gravidarum, induced by hormone secretion changing. Secondly, women may worry about the condition of spontaneous abortions and abnormal fetal development more in first trimester In addition, pregnant women might face the risk of malformations with first trimester exposure in the terrible living environments such as second-hand smoke, radiation, alcohol, etc [27]. Nevertheless, in the second trimester, pregnant women are more aware of the fetal health status after a series of physical examinations. At the same time, pregnant women adapted better to their new role. We found the incidence of depression during pregnancy of this study is higher than previous study [27]. One possible explanation is that two studies used different scale (EPDS vs. Hospital Anxiety and Depression Scale) to assess probable depression. Besides, the differences finding between the two studies may be due to economic and cultural differences, that Hong Kong is a developed city in China and Chengdu is a developing city in mainland China.

The total incidence of depression during pregnancy was 45.67% in current study which was higher than most cross-sectional studies in China. The reason may because this study was a longitudinal research addressing the prevalence of antenatal depression in all trimesters of pregnancy instead of only one trimester. Also, the prevalence of each trimester was higher than the research conducted by Lee [26]. It illustrated that there were new cases or recurrent cases in the second and third trimester of pregnancy. The probable reason might be developing antenatal depression involving multiple factors, for instance, recent major life events, which were in line with previous researches [28]. It is recommended that conducting multiple times assessments about depression to assess the mental status of pregnant women throughout pregnancy. This would be benefit for health of baby and the mental health of pregnant women who are susceptible to depression by enabling appropriate interventions.

### Family support (marital satisfaction, and harmony with parents-in-law) protect pregnant women from depression

Multivariate analysis among the medium or high risk group and the low risk group illustrated that the good partner relationship and the good relations with parents-in-law were the protective factors for both medium and high risk group, which correspond to the general idea that spouse’s relationship is the foundation of a family. The marriage surviving is inseparable from harmony relationship in the modern society with high divorce rate [29]. Couples should have the consciousness that giving birth to a baby is a conjunct responsibility of couples instead of wife’s own. A understanding, supportive and careful partner could help pregnant women getting through the period of pregnancy smoothly. One study demonstrated that stronger marital relationship can deeply affect the mental health of pregnant women [30]. However, in reality, people getting married not only for love, sometimes because they were “supposed” to. Or, they get married before they really know that person. Thus, the controversial issues make couples struggling lack of support and tolerance. It is a fact that women experience hormonal changes, altered body image during pregnancy, besides they may worry a lot about health of the baby. All these factors could affect the mood of the pregnant women while the mood change are more likely to affect the marital relationship. Relationship can, in turn, lead to more stress on the mental health of the pregnant, and a vicious circle ensues. In addition, health care provider might recommend avoiding sex in the first and third trimesters which might affect the marital satisfaction and intimacy [31]. The debate about the different expectations in terms of desired sex of the baby, delivery mode, living status and education of the baby may be another issue challenge the couples. Thus, pregnancy actually is a stress event for both husband and wife. The relations between parents-in-law and daughter-in-law, a unique and miracle issue in China, also affect the marital relationship [32, 33]. Good relations with parents-in-law can promote a more harmonious relationship between husband and wife, and vice versa. Therefore, to prevent pregnant women from depression or to improve the their depression, health care provider could ask family members to provide more support, such as accompany pregnant women to attend prenatal health examination, and maternal education classes knowing more about diet during pregnancy, pregnancy risk prevention, delivery mode, the Chinese tradition of “doing the month”, breast-feeding and neonatal care, etc. Couples should be encouraged to cultivate common hobbies and participate in social activities together. Having sex during pregnancy won't provoke a miscarriage. Most miscarriages occur because the fetus isn't developing normally. If sex is difficult, or off-limits, try cuddling, massage or kissing. Couples should be encouraged to learn marriage emotion courses, or conduct professional marriage counseling to improve the relationship between husband and wife. Finally, couples should pay attention to the relations between pregnant women and their parents-in-law, keep family harmony, obtain strong family support to ensure the health of mothers and infants.

### Cultivating the confidence of pregnant women in delivery, and reducing the impact of psychological vulnerability on pregnant women with depression

The multivariate analysis showed that fear of dystocia was the risk factor of pregnant women be depressed in the medium and high-risk group. This might be explained by the character of psychological vulnerability. People who are psychological vulnerable are prone to worry, anxiety or depression about uncertain events. Pregnancy and childbirth are one important but dangerous process in women's life. Although childbirth is much more safe than past, pregnant women may still possible confront with unexpected dangerous, for instance, emergency cesarean section, dystocia, etc. Similar to the results of the study carried out by Song [34], most pregnant women worry about emergency cesarean section from vaginal delivery and dystocia. For the primiparas, most of the information about childbirth are from their friends, books or media. It is hard for them to exactly distinguish the reliable information. This might lead to their fear of dystocia and lack of confidence in childbirth. Although vaginal delivery is the most common and natural type of delivery method, which are usually preferable for both mothers and babies. Pregnant women might be under stress if she received too many suggestions from others such as family members and health care providers.

Therefore, pregnant women and their family are encouraged to participate in maternal education classes, and obtain relevant knowledge such as pregnancy health care, delivery, etc. The recommendations related to the appropriate types of exercise are likely to be made. They might experience Doula labor. Secondly, family members and medical workers need to respect the pregnant women's choice of delivery methods.

## Conclusion

Depression can occur in any stages of pregnancy. The incidence and levels of depression in the first trimester of pregnancy were the highest among the three trimesters of pregnancy. Therefore, it is important to enhance screening of depression in first trimester and enable appropriate interventions as early as possible. The results of current study suggested that it is necessary to establish a good partner relationship and good relations with parents-in-law, to have the confidence of childbirth, to reduce the impact of negative events on pregnant women, so as to improve the symptom of depression during every stages of pregnancy and promote the well-being of mothers and children.

## Limitations

The current study had some limitations, first, some items such as the marriage satisfaction was measured by a subjective evaluation instead of accurately measured. In the future study, an objective investigation scale should be recommended. Secondly, the research was limited by the possible selection bias introduced by numbers of women lost to follow up. Although we used the scientific method of multi stage stratified sampling to recruit pregnant women from four hospitals at different levels in Chengdu. But in a way, attrition is inevitable in longitudinal epidemiological studies. Bellón’s [35] multi-center longitudinal study of predictors of attrition of major depression in primary care showed 1638 (30%) patients at 6 months and 1875 (34%) patients at 12 months did not follow up, and sociodemographic factors were strongly associated with attrition. Another multi-center longitudinal research of Depression and Anxiety showed that younger age, less years of education, having major depressive disorder and comorbid depressive and anxiety disorders etc. were determinants of attrition [36]. These factors may lead to a decrease in the incidence of depression. In future research, we need to pay attention to the characteristics of the attrition. According to the different associated factors of attrition, a more scientific retention strategies should be applied to decline the loss of samples. In addition, this study was conducted in Chengdu may not be a represented sample of China. In future, national survey are needed to know more about pregnant women. Furthermore, according to Hawthorne effect, pregnant women may modify their behavior simply because they are being observed, a more implicit way detect depression are needed in the future.

## Data Availability

The datasets used and analyzed during the current study are available from the corresponding author on reasonable request.

## Abbreviations

AD: Antenatal depression
EPDS: Edinburgh Postnatal Depression Scale
OR: Odds ratios
CI: Confidence interval
